# Acute Left Internal Carotid Artery and Right Popliteal Artery Occlusion Related to Cisplatin-Gemcitabine Based Chemotherapy

**DOI:** 10.1155/2018/9281918

**Published:** 2018-12-17

**Authors:** Saman Zafar, Rashmika Potdar, Andrew Tiu, Gabor Varadi, John Leighton

**Affiliations:** ^1^Department of Neurology, Einstein Medical Center, Philadelphia, USA; ^2^Division of Hematology and Medical Oncology, Einstein Medical Center, Philadelphia, USA; ^3^Department of Medicine, Einstein Medical Center, Philadelphia, USA

## Abstract

**Objectives:**

The increased risk of thromboembolic complications with active cancer is well known. We present this case to highlight that chemotherapy may increase the risk of thromboembolic events even further in cancer patients.

**Methods:**

We report a case of a 64-year-old male with Diffuse Large B-Cell Non-Hodgkin's Lymphoma who presented with left-sided headache and right calf pain two weeks after starting Rituximab/Gemcitabine/Cisplatin/Dexamethasone chemotherapy. Neurological examination was normal, but there was an absent right dorsalis pedis pulse. He subsequently developed left vision loss. CT angiogram of the head and neck revealed occlusion of his left internal carotid artery and poor opacification of the left ophthalmic artery. An angiogram of the right leg further revealed acute occlusion of the popliteal artery.

**Results:**

The patient underwent intra-arterial Tissue Plasminogen Activator injection to his lower limb and was started on Low Molecular Weight Heparin. His vision gradually recovered with time. His chemotherapy regimen was changed to RICE (Rituximab, Ifosfamide, Carboplatin, Etoposide).

**Conclusion:**

Based on literature review, there are numerous similar presentations of arterial thromboembolism in patients on Cisplatin-based chemotherapy. A high index of suspicion for such events should be maintained for patients on chemotherapy presenting with unusual symptoms.

## 1. Case Description

A 64-year-old male with Diffuse Large B-Cell Non-Hodgkin's Lymphoma presented with one week of left-sided headache and right anterior calf pain, which worsened with walking and improved with rest. He also described low-grade fever and fatigue.

His past medical history was significant for Mucosa-Associated Lymphoid Tissue (MALT) Lymphoma, previously treated with Rituximab. However, within the past two years, he gradually developed breathlessness and was found to have hilar lymphadenopathy. Further work-up had led to a diagnosis of Diffuse Large B-Cell Lymphoma. This had initially been treated with Rituximab, Cyclophosphamide, Doxorubicin, Vincristine, Prednisone (RCHOP) chemotherapy. He progressed approximately one year later with mediastinal lymphadenopathy. At this point, he was considered a candidate for high-dose therapy with stem cell transplant if induced into remission and, with this intention, had started salvage chemotherapy with Rituximab/Gemcitabine/Cisplatin/Dexamethasone. A week after starting this chemotherapy regimen, he developed the headache and claudication that led to this hospitalization.

He did not have any other past medical problems. There was no history of previous cardiovascular disease and no family history of premature cardiovascular disease. He was an ex-smoker and did not have substance abuse issues.

On examination, he had normal cardiovascular, neurological, pulmonary, and gastrointestinal examination. He did, however, have an absent right dorsalis pedis pulse.

### 1.1. Investigations

**Figure 1:**
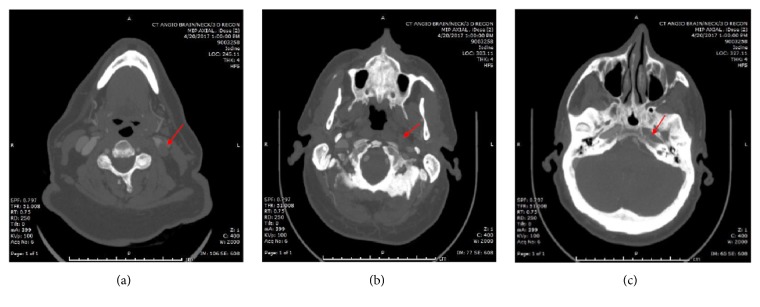
CT angiogram head and neck. Note the occlusion of the left internal carotid artery, starting at the level of the common carotid bifurcation (a), in the neck (b), and in the carotid canal (c).

**Figure 2:**
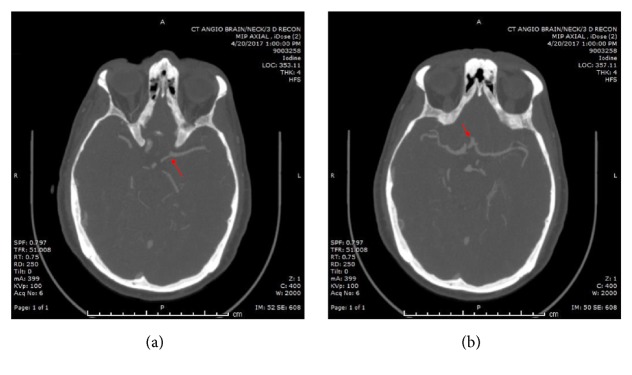
CT angiogram head and neck (continued): note the absence of the left internal carotid artery intracranially and poor opacification of the left ophthalmic artery. There is collateral supply to the left middle cerebral artery from the left posterior cerebral artery (a) and the anterior communicating artery (b). An intact circle of Willis allowed the left middle cerebral artery to maintain supply despite the complete occlusion of the left internal carotid artery.

Initial Computed Tomography (CT) Scan of the head and Duplex Scan of the leg were normal. His Magnetic Resonance Imaging (MRI)/Magnetic Resonance Angiography (MRA) Brain revealed no acute stroke and no intracranial atherosclerosis. Within twenty-four hours of hospital admission, he developed a visual defect in his left eye. CT angiogram of the head and neck was done and revealed occlusion of his left internal carotid artery, poor opacification of the left ophthalmic artery, and the presence of good collateral flow from the left posterior cerebral artery and the anterior communicating artery (Figures 1 and 2). There was no evidence of atherosclerosis elsewhere. Headache is an unusual symptom of acute internal cerebral artery occlusion, but dissection and vasculitis were excluded based on the CT angiogram of the head and neck.

**Figure 3:**
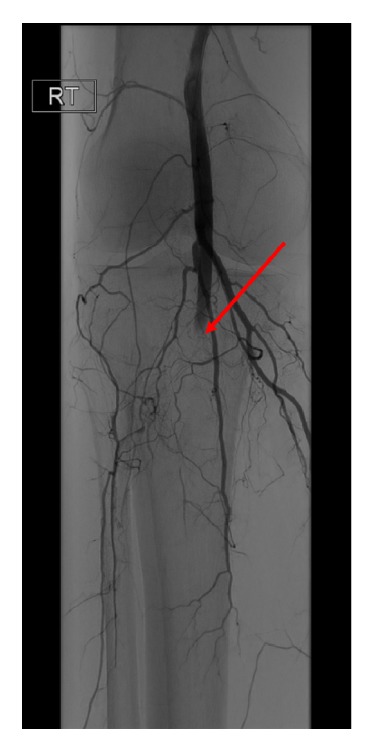
Right lower extremity angiogram demonstrating acute occlusion of the distal popliteal artery with no significant runoff vessels. A catheter was placed in this artery and Tissue Plasminogen Activator (TPA) was infused overnight. Subsequent evaluation revealed resolution of thrombus within the popliteal artery and tibioperoneal trunk.

An angiogram of the right leg revealed acute occlusion of the distal popliteal artery (Figure 3).

Given the absence of stroke and the presence of good collateral flow in the brain, it was initially unclear whether the occlusions of his left carotid artery and right popliteal artery were acute or chronic.

Cardioembolism was excluded by a normal transthoracic echocardiogram, and telemetry monitoring did not reveal atrial fibrillation.

The differential diagnosis for these arterial thromboembolic events included acute disseminated intravascular coagulation (DIC), thrombotic thrombocytopenic purpura (TTP), and antiphospholipid antibody syndrome. Laboratory results and peripheral blood smear excluded these conditions.

### 1.2. Subsequent Course

Because of the visual defect in the left eye, the ophthalmologist was called and found the patient to have a left ophthalmic artery occlusion, embolic in nature. With new-onset vision loss secondary to embolization from the left carotid artery thrombus and the acute nature of all his symptoms, the left carotid artery thrombosis was also favored to be acute. After review of medical literature, we discussed whether Cisplatin/Gemcitabine had any role to play in the pathogenesis of his acute arterial thromboembolic events.

### 1.3. Management

Initial management with antiplatelet and anticoagulant therapy had to be delayed because of the thrombocytopenia (25,000/mcl) he was experiencing because of his recent chemotherapy. After platelet transfusions to treat his thrombocytopenia, he underwent intra-arterial Tissue Plasminogen Activator (TPA) injection by Vascular Surgery to the right popliteal artery.

As his platelet count increased, he was initiated on Low Molecular Weight Heparin treatment and discharged home. His left vision gradually recovered with time.

Moving forward, a decision was made to avoid the thrombogenic potential of Gemcitabine and Cisplatin. His chemotherapy regimen was changed to RICE (Rituximab, Ifosfamide, Carboplatin, Etoposide).

## 2. Discussion

Patients with cancer face a substantially increased short-term risk of arterial thromboembolism [1]. There is, however, less awareness that part of this risk may be related to the chemotherapy that patients are receiving, rather than the hypercoagulable state induced by the cancer itself.

Recently, several case reports have implicated Cisplatin with different forms of arterial thromboembolism [2]. Cases like ours with carotid artery occlusion [3, 4] or peripheral arterial occlusion leading to limb ischemia, gangrene, and amputation [5, 6] are reported. Other presentations that have been reported are stroke [7], myocardial infarction [8], abdominal aorta occlusion [9], and renal artery thrombosis leading to renal necrosis [10].

The role of Gemcitabine in causing arterial occlusions is less well established, with conflicting reports in the literature about its relationship to thrombotic microangiopathy or thromboembolism [11].

The combined effect of Cisplatin and Gemcitabine in causing thromboembolic events is infrequently reported, given the widespread use of these chemotherapeutic agents in a variety of cancers [12]. A study on Non-Hodgkin's Lymphoma patients treated with Gemcitabine, Dexamethasone, and Cisplatin (GDP) showed that 7/51(14%) developed thromboembolic episodes, including 1 fatal pulmonary embolism and 1 asymptomatic abdominal aortic thrombosis [13]. Valle et al. compared the role of Gemcitabine alone with Cisplatin and Gemcitabine in cholangiocarcinoma; the rate of thromboembolic events increased from 3/199 (1.5%) to 7/198 (3.5%) [14].

Our patient did not have any cardiovascular risk factors apart from prior smoking history. His symptoms all started after initiation of chemotherapy, and during workup, no atherosclerosis or cardiac source of his carotid or tibial artery occlusions was identified. He had a history of two previous chemotherapy treatments with no complications; the only new drugs in his present regimen were Cisplatin and Gemcitabine. Based on the present literature, it seemed reasonable to assume that his new chemotherapeutic medications may have increased his risk of arterial thrombosis, and his regimen was changed.

## 3. Conclusion

Arterial occlusion in cancer patients can cause a variety of puzzling symptoms and a high index of suspicion should always be present for ordering an appropriate scan to exclude arterial occlusion. Flank pain should prompt concern for renal or splenic infarction, leg pain for aortic or peripheral arterial occlusion, neurological symptoms for stroke, chest pain for myocardial infarction, among other thrombotic syndromes. Increasing awareness will lead to timely diagnosis and prevention of devastating consequences for the patient.

It is important to increase the awareness about the risk of arterial occlusion and vascular events with Cisplatin-based chemotherapy, as this facilitates a better understanding of the patient's disease process and allows for improved future planning of care.
